# Fungal periprosthetic joint infection following total elbow arthroplasty: a case report and review of the literature

**DOI:** 10.1186/s13256-016-1176-0

**Published:** 2017-01-21

**Authors:** Cory A. Kwong, Shannon K. T. Puloski, Kevin A. Hildebrand

**Affiliations:** 1 Orthopaedic Surgery Resident PGY-3, Section of Orthopedic Surgery, Department of Surgery, University of Calgary, Health Sciences Centre, 3330 Hospital Drive NW, Calgary, AB T2N 4N1 Canada; 2 Section of Orthopedic Surgery, Department of Surgery, University of Calgary, Health Sciences Centre, 3330 Hospital Drive NW, Calgary, AB T2N 4N1, Canada; 3 Department of Surgery, University of Calgary, Health Sciences Centre, 3330 Hospital Drive NW, Calgary, AB T2N 4N1, Canada

**Keywords:** Total elbow arthroplasty, Periprosthetic joint infection, *Aspergillus terreus*, Infection, Rheumatoid arthritis, Fungal, Revision, Resection arthroplasty

## Abstract

**Background:**

With improving surgical techniques for total elbow arthroplasty clinical outcomes have improved and its utilization continues to increase. Despite these advances, complication rates remain as high as 24%. Of these complications periprosthetic joint infection is one of the most common and morbid. The rheumatoid elbow remains a leading indication for total elbow arthroplasty. Patients with this condition frequently require immunosuppressive therapy, which places them at higher risk of both typical and atypical infections.

**Case presentation:**

We present the case of a persistent, late-onset periprosthetic joint infection in a total elbow arthroplasty of a 64-year-old Caucasian woman with severe refractory rheumatoid arthritis. The offending pathogen, *Aspergillus terreus*, is previously unreported in the arthroplasty literature and grew concurrently with coagulase-negative staphylococcus. Eradication of the fungal and bacterial agents involved resection arthroplasty, serial debridement, and multiple courses of intravenous and oral antimicrobial therapy. Two attempts at reimplantation arthroplasty failed to eliminate the infection and our patient ultimately required definitive resection arthroplasty.

**Conclusions:**

Arthroplasty in the rheumatoid elbow confers with it a high complication rate. Inflammatory disease and immunosuppressive drugs combined with the subcutaneous anatomy of the elbow contribute to the risk of infection. Fungal periprosthetic joint infection in the rheumatoid patient presents both diagnostic and therapeutic challenges. Fungal growth should always be treated and requires organism-specific antimicrobials in conjunction with surgical debridement. More literature is needed to determine the optimal treatment regimen for this devastating complication.

## Background

With improvements in outcomes of total elbow arthroplasty (TEA) [1], the procedure has become increasingly more common. Rates of TEA utilization in the United States doubled over a 14-year period to a rate of 0.96 per 100,000 [2]. Despite advances in technique and hardware components, complication rates remain high in comparison to other joint arthroplasties. A systematic review of the literature from 1992 to 2009 estimated that the significant complication rate was as high as 24.3% [1]. Of these complications, periprosthetic joint infections (PJI) are one of the more common and most devastating, and have been estimated to occur in 5–8% of patients [3]. The treatment of PJI is well described in arthroplasty literature and carries with it significant morbidity. However, the literature on PJI in TEA is still limited and, to the best of our knowledge, there are no published reports of fungal infection following TEA. The existing literature concerning fungal PJI of the other joints mostly consists of case series and consensus statements. Here we present the complex case of persistent, late-onset Aspergillosis of a total elbow arthroplasty in a patient with severe refractory rheumatoid arthritis.

## Case presentation

A right-handed, 64-year-old Caucasian woman presented for reimplantation left TEA. She had had a left total elbow resection arthroplasty in April 2014 due to a fungal PJI. Her past medical history was significant for a 41-year history of severe refractory rheumatoid arthritis involving multiple joints and cervical spine. She had failed multiple medical therapies including disease-modifying antirheumatic drugs and biologics, and was treated intermittently with prednisone for flares.

**Fig. 1:**
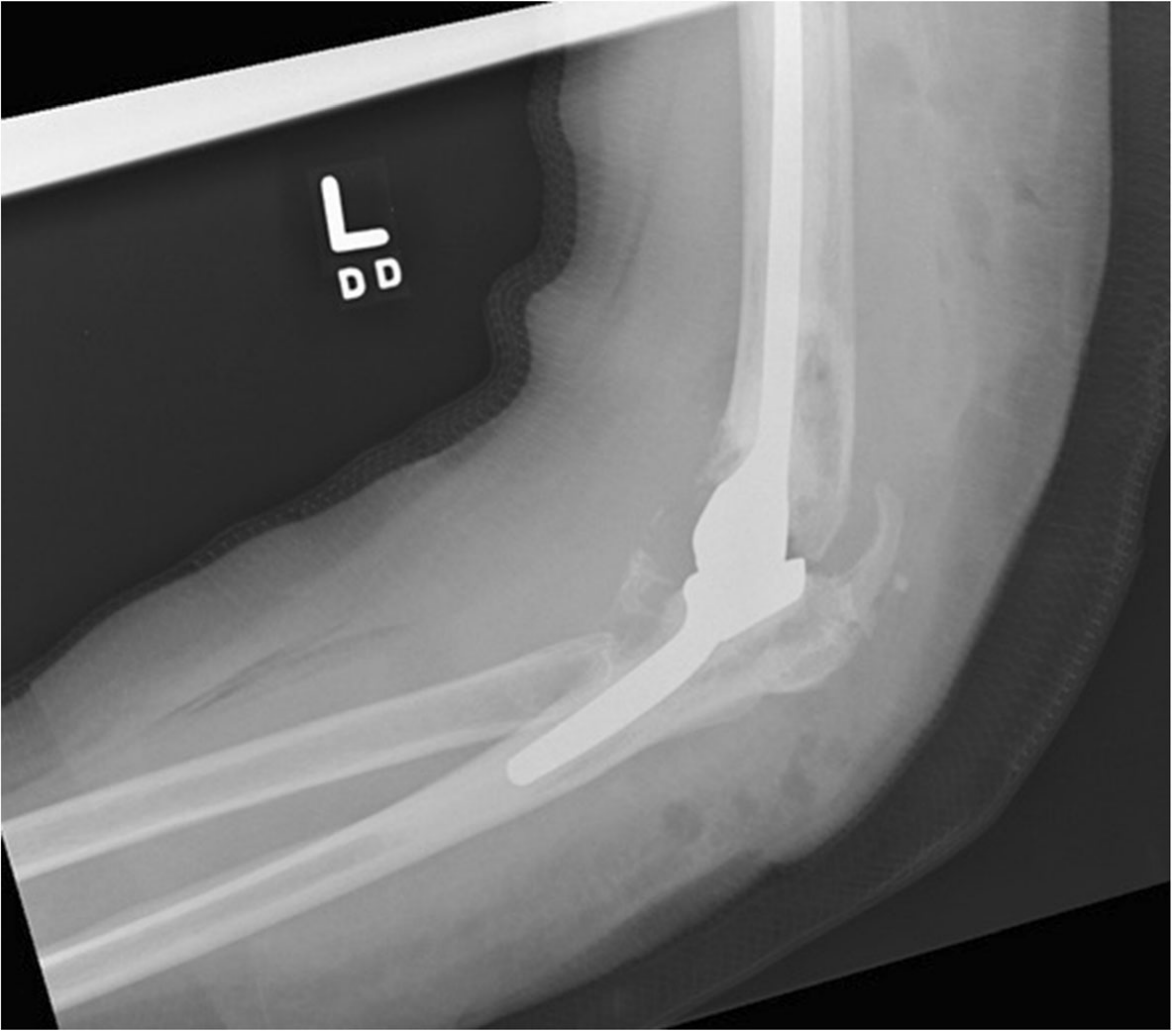
Left total elbow arthroplasty showing well-fixed components

The original DePuy Pritchard TEA (DePuy, Warsaw, IN, United States) was implanted in 1995. It had performed well until June 2007 when she developed left elbow pain and fevers and received a liner exchange in well-fixed implants (Fig. 1). The TEA was retained until she required resection arthroplasty in July 2011 after repeat debridement and antimicrobial regimens failed to resolve a draining sinus due to a coagulase-negative staphylococcus (CONS) infection. The implant was replaced with a vancomycin-impregnated cement spacer. Intraoperative cultures grew CONS for which she was treated with cefazolin for 8 weeks. Two months postoperatively the spacer was removed but tissue cultures remained positive for polymicrobial infection including CONS and *Enterobacter cloacae*. Extended antimicrobials consisted of 6 weeks of ciprofloxacin and vancomycin. In December of 2011 reimplantation was attempted but abandoned when intraoperative frozen sections showed >30 white blood cells per high-powered field (WBC/hpf). Definitive tissue cultures grew new *Aspergillus terreus* only, which was thought to be a contaminant after growing on only one of six fungal cultures.

**Fig. 2:**
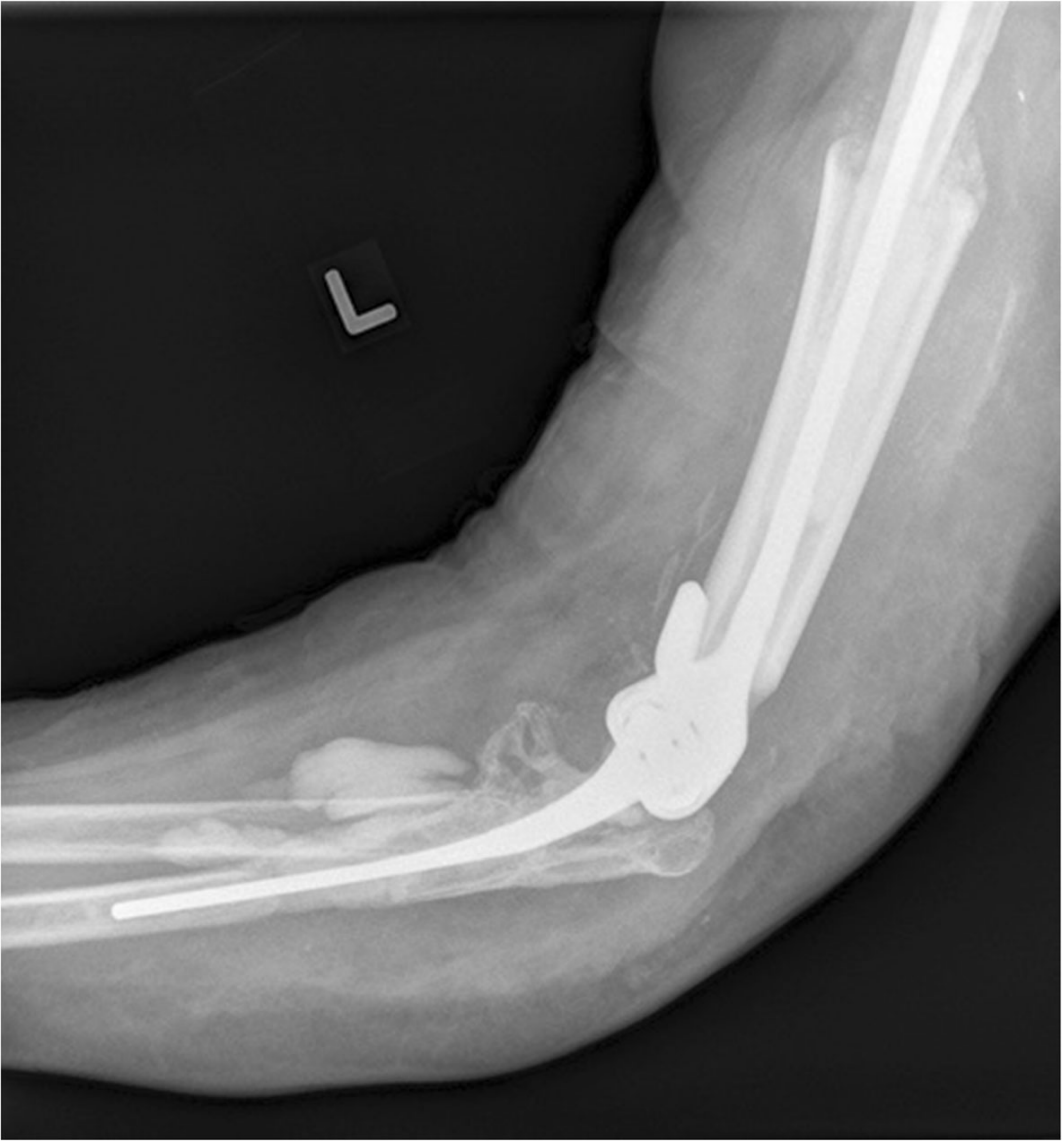
Left revision total elbow arthroplasty with cemented allograft prosthetic composite

In February 2012, she was off of all immunosuppressives and systemically well. The elbow was healed and free of any drainage. Her C-reactive protein (CRP) level was 8.7 mg/L and erythrocyte sedimentation rate (ESR) was 29. Reimplantation surgery was undertaken using a similar method reported by LeBlanc *et al*. 2012 [4]; an allograft-prosthetic composite was used consisting of a long-stemmed cemented Coonrad-Morrey TEA (Zimmer) and tibial allograft on the humeral side (Fig. 2). Intraoperatively there was no concern for infection, but final cultures grew scant CONS and *Aspergillus terreus* and histologic samples were nonspecific. She was treated as a mixed fungal and bacterial PJI and treated with 8 weeks of intravenous voriconazole and vancomycin. The implant was retained.

**Fig. 3:**
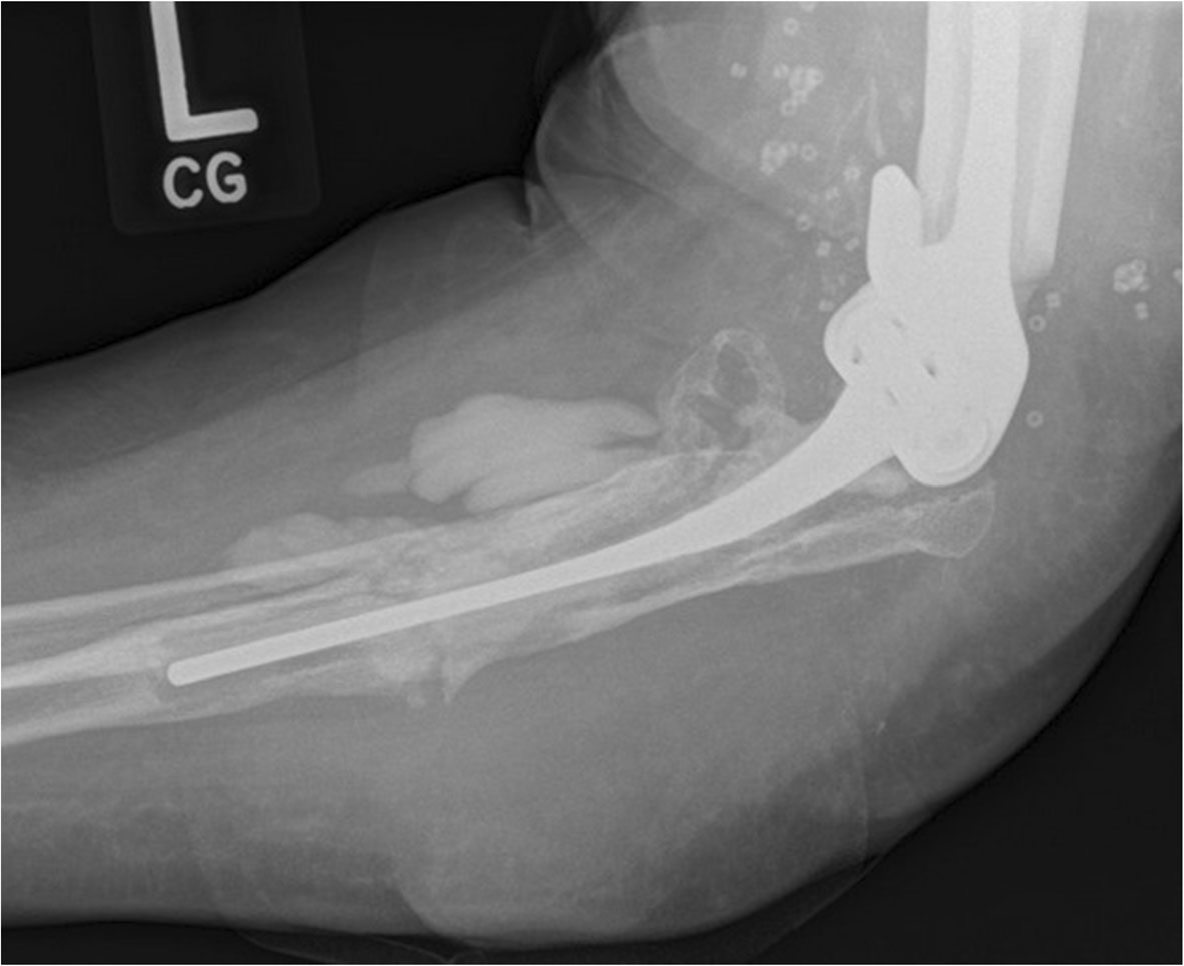
Revision total elbow arthroplasty with new ulnar-sided insufficiency fracture

In the following 6 months she healed and was doing well until a rheumatoid flare. She was put on a trial of abatacept and shortly after developed a fluctuant mass around the lateral distal humerus. An aspiration of the collection grew *Aspergillus terreus* and the abatacept was stopped. Blood work showed a CRP level of 10.4 and an ESR of 50. Over the course of the next 7 months, she would undergo eight more serial aspirates of the recurrent collection with only the first specimen growing *Aspergillus terreus*. A bone scan showed no increased uptake, but repeat radiographs showed the presence of an insufficiency fracture on the ulnar side (Fig. 3). During this time she remained off of antimicrobial treatment in an attempt to identify the offending organism.

With failure to control Aspergillosis of the left TEA with combined serial (two) operative debridements and medical management, a second resection arthroplasty was performed in April 2014. *Aspergillus terreus* grew on one of three cultures but no bacterial growth was identified. Postoperative antimicrobial therapy consisted of caspofungin and cefazolin for 8 weeks, followed by cephalexin and a short trial of oral voriconazole, which was stopped due to gastrointestinal intolerance and transaminitis.

**Fig. 4:**
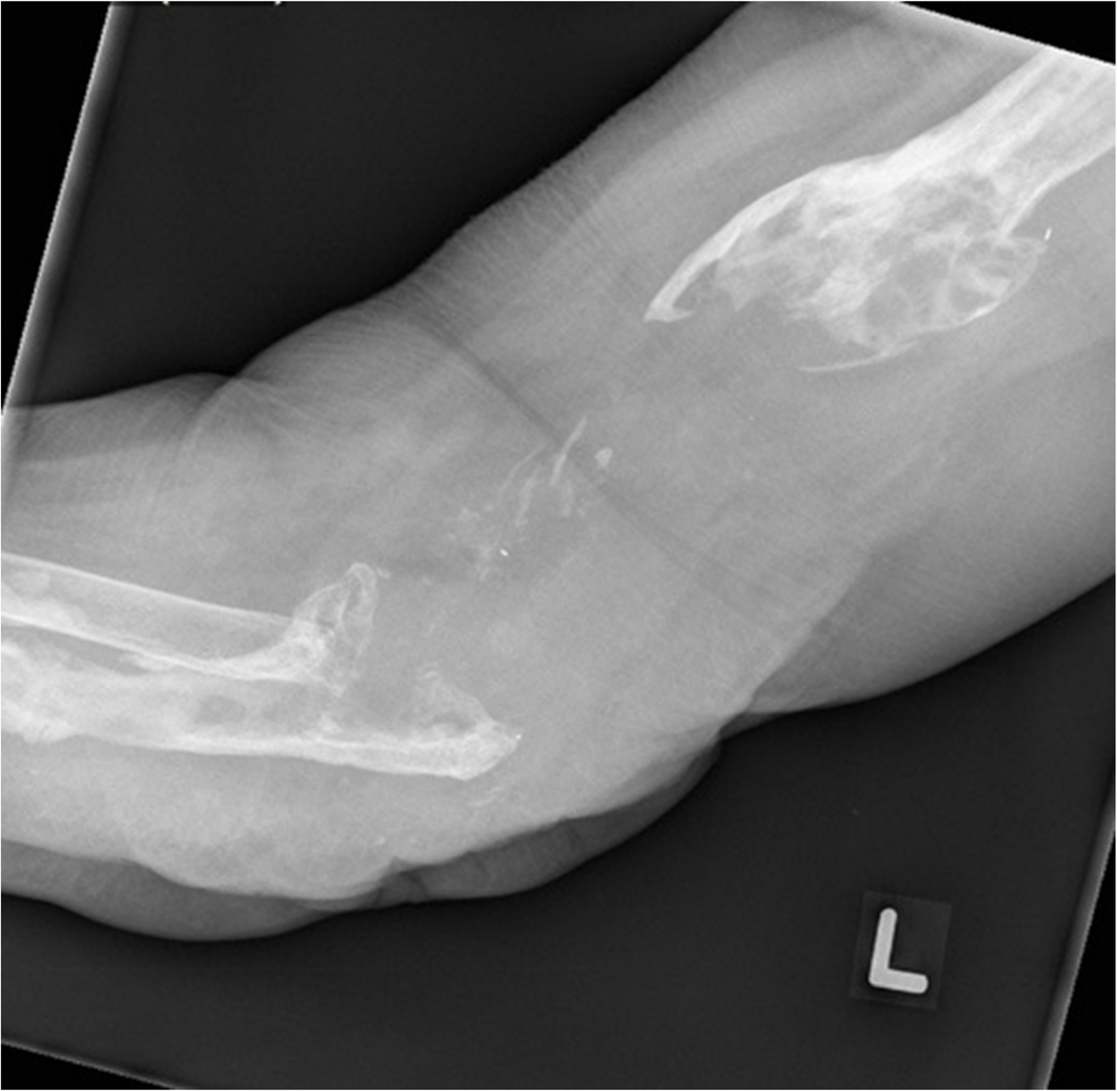
Left elbow resection arthroplasty

Eleven months post left TEA resection arthroplasty for chronic Aspergillosis, she was systemically well and had been off of antimicrobials for 5 months. A physical examination revealed a left flail elbow, she was neurologically intact with good function of the hand and a well-healed posteriorly based scar. There were no signs of infection or inflammation. Her most recent laboratory test results showed a WBC count of 6.2 × 10E9/L, a CRP level of 2.2 mg/L and an ESR of 25, all within normal limits. The last positive tissue culture was from the resection arthroplasty in April 2014 and no further specimens had been collected. Plain radiographs showed a significant osseous defect of the proximal ulna and distal humerus (Fig. 4). A bone scan and white blood cell scan showed no definitive evidence of ongoing infection.

**Fig. 5:**
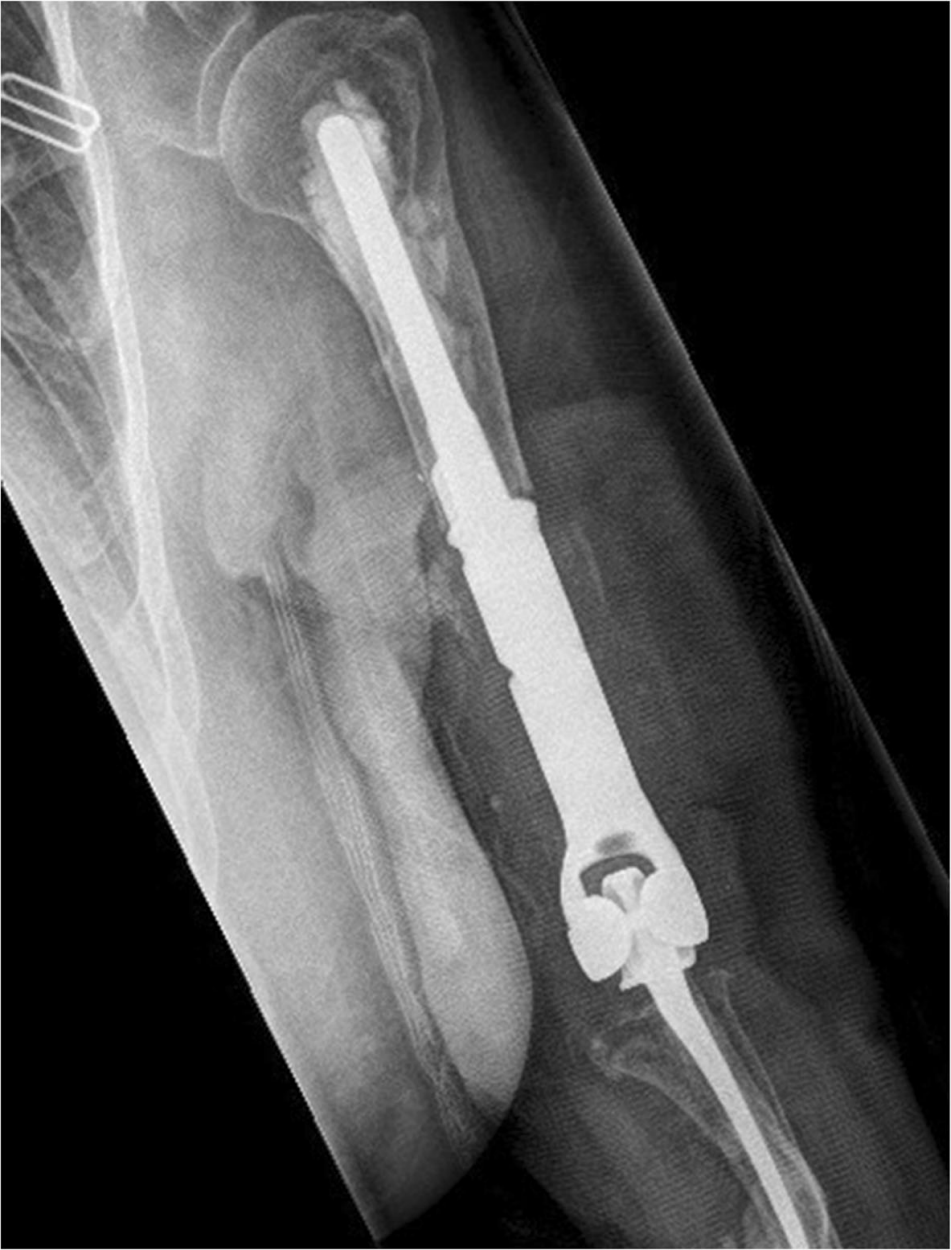
Left revision total elbow arthroplasty anteroposterior view

**Fig. 6:**
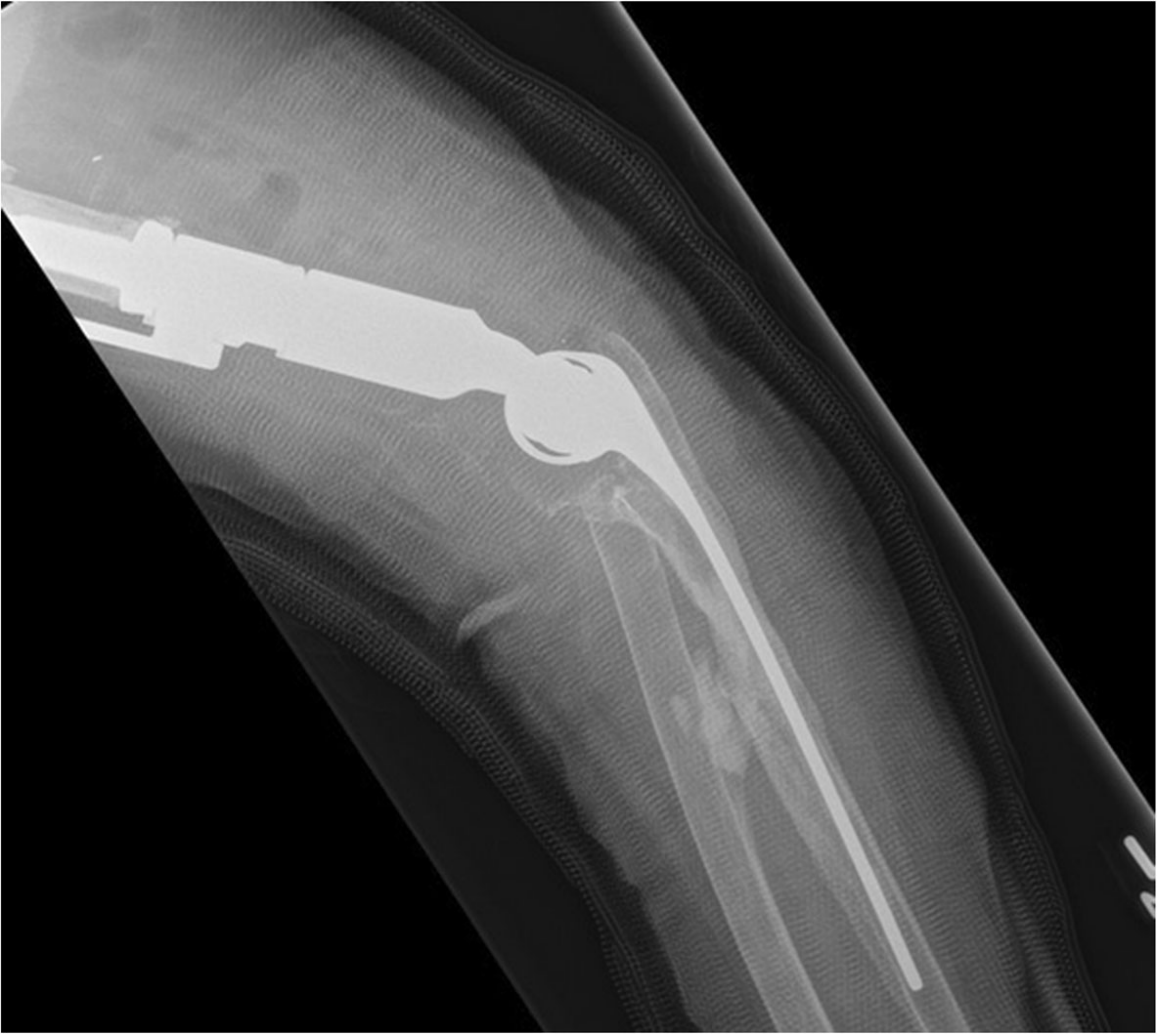
Left revision total elbow arthroplasty lateral view

In March 2015, after consultation with infectious diseases and local orthopedic colleagues, our patient and surgeons elected to proceed with a second attempt at reimplantation arthroplasty. Due to extensive bone loss, the reimplantation was performed using a cemented distal humerus endoprosthesis and long-stemmed ulna component (Biomet SRS/Discovery TEA; Zimmer Biomet) (Figs. 5 and 6). Multiple tissue specimens collected intraoperatively were negative for fungal and bacterial growth.

Unfortunately, after a brief symptom-free period she developed a recurrent sinus over the tip of the olecranon and recurrent CONS was confirmed. Our patient has since undergone definitive resection arthroplasty. No further fungal infection was identified and no further surgery is planned.

## Discussion

### Fungal infection in elbow arthroplasty

Only 1% of PJIs are of fungal origin [5], and because of this rarity, the literature is limited to a few small case series and consensus statements mostly based on hip and knee arthroplasty. Since relatively few TEAs are performed compared to hip and knee arthroplasties, the incidence of fungal PJI in TEA is unknown [6]. The most common organisms in fungal PJI are Candida species [7]. *Aspergillus fumigatus* and *Aspergillus niger* have also been reported in the arthroplasty literature in small numbers [8] but, to the best of our knowledge, there have been none for *Aspergillus terreus*.

Many patients requiring TEA have pre-existing rheumatoid arthritis (RA). It has been proposed that the high complication rate of TEA in patients with RA could be attributed to a combination of poor bone stock, minimal posterior soft tissue coverage, and immunodeficiency [9]. In RA, either the pathologic process itself causes the immunodeficiency, or the pharmacology used to treat it [10]. Like in our case, many of these patients will be at higher risk of atypical infections, which can potentially lead to catastrophic morbidity

### Diagnosis of fungal infection in elbow arthroplasty

Fungal PJIs are often hard to diagnose and can be complicated by comorbidities and concurrent or previous bacterial infection [10]. Regardless, diagnosis begins with a thorough history and physical examination similar to any presentation of PJI. Diagnostic imaging including plain radiographs should always be obtained, but advanced three-dimensional imaging and nuclear medicine tests have not been recommended for routine use in the diagnosis of PJIs [11]. Serologic markers are unable to distinguish between causative organisms and synovial fluid rarely identifies fungal pathogens [10]. As a result of this, special attention must be given to specimen collection, as routine cultures may show no growth in the setting of a high clinical suggestion. To improve diagnostic yield, serial joint aspirations and multiple intraoperative specimens from diagnostic or therapeutic procedures are essential to help establish the causative organism [12, 13]. Fungal cultures should be plated on fungal selective media (for example, Sabourad dextrose) and growth can take up to 4 weeks [5]. When cultures do yield fungal organisms, results are still often misinterpreted. In a 2013 systematic review of fungal PJIs, Kuiper *et al.* found that fungal growth was initially considered contamination in 21% of cases. They concluded that any fungal species grown should thus be considered a pathogen [7].

In this case, the underlying RA and history of bacterial PJI in the same joint complicated the diagnosis. Serologic markers including ESR and CRP were difficult to interpret in the context of severe RA. The previous and intermittent presence of CONS from aspirates and surgical specimens not only confounded organism-specific therapy but also significantly increased the patient’s baseline risk for a fungal PJI [12]. Initial suggestion of *Aspergillus* spp. as a contaminant may have led to delayed time to antifungal treatment and premature second-stage reimplantation with the first revision. In retrospect, a histologic evaluation of WBC/hpf at the time of reimplantation in February 2012 may have detected continued inflammation and reconsideration of the *Aspergillus* spp. as a contaminant. However, while frozen histologic section has been shown to be highly specific at 93.1%, it is only 51.3% sensitive [14]. Additionally, its utility in both fungal infections and patients with underlying arthropathies is still poorly defined [15].

### Eradication of fungal infection in elbow arthroplasty

Fungal PJIs are not only difficult to diagnose but are also thought to be challenging to treat. Standardized protocols for the treatment of PJI have been produced by the Infectious Diseases Society of America (IDSA) but have not been customized for fungal PJI, let alone fungal PJI in TEA. The optimal treatment of PJI consists of both medical and surgical intervention [3, 11, 12].

Medical management includes systemic antifungals prior to reimplantation, and debate still exists surrounding the use of antifungal-impregnated spacers owing to a lack of evidence [12]. The IDSA currently recommends between 4 and 6 weeks of organism-specific intravenous or highly bioavailable oral antimicrobial therapy following resection arthroplasty, but does not distinguish between bacterial and fungal organisms. As was seen in this case, prolonged use of antifungals raises the risk of systemic toxicity and poses a challenge when considering length of therapy [5]. Cheung *et al*. used a similar antimicrobial treatment regimen in their experience of 29 bacterial PJIs of TEAs. Medical management in two-stage revisions included tobramycin/vancomycin-impregnated cement spacers and 6 weeks of organism-specific intravenous antibiotics. None of their causative organisms were fungal [16].

Surgical options include resection arthroplasty, one- and two-staged revision, arthrodesis, and amputation. A 2015 systematic review of 45 fungal infections in total knee arthroplasty recommended a two-staged approach as the gold standard. This consisted of resection arthroplasty with or without antibiotic-impregnated cement spacers, followed by delayed reimplantation. As an initial surgical intervention, the failure rate was approximately 30% [12]. Cheung *et al*. reported on 29 TEA reimplantations for bacterial PJI from 1976 to 2003. They showed a similar failure rate of 28%, with a 3-year survival of 77% and 8-year survival of only 48% [16]. The necessity for removal of all original hardware may be attributable to the ability of fungi, including *Aspergillus* spp., to form hardy biofilms overlying prostheses [17]. A 1998 retrospective study on PJI in TEA showed that four of four *Staphylococcus epidermidis* PJIs, a known biofilm-forming species, failed irrigation and debridement with hardware retention, compared to two of eight failures for *Staphylococcus aureus* PJIs [18].

## Conclusions

Fungal PJIs in TEA are different from both bacterial PJIs in TEA, and fungal PJIs in hip and knee arthroplasty. Host risk factors including inflammatory disease and immunosuppressive drugs combined with the subcutaneous anatomy of the elbow are possible risk factors that could contribute to the higher complication and infection rates.

It has been hypothesized that the introduction of modern antirheumatic drugs has contributed to a decrease in the utilization of joint surgery in rheumatoid arthritis [17]. Even though improved medical treatment in RA may have contributed to a decreased incidence of rheumatoid elbow as an indication for TEA [6], patients who do undergo the procedure for this indication may be at higher risk of atypical PJIs caused by fungi.

A lack of literature on this rare but morbid complication left the responsible team without a precedent on which to base treatment. In this case, fungal infection of a TEA for rheumatoid elbow proved to be an extremely difficult complication to manage and caused considerable morbidity to the patient. Owing to the rarity of this complication, demonstrated by the lack of literature, future cases will pose similar therapeutic dilemmas. If presented with a similar case, the authors advise orthopedic surgeons and infectious disease specialists not to attribute positive fungal cultures to contamination, especially when it may delay treatment in an immunocompromised host. As was seen in this case, fungal infection may persist despite evidence of preoperative sterility. In patients with a history of recurrent infected TEA, surgeons should be wary of the morbidity of multiple attempts at revision endoprosthetic reconstruction in favor of earlier resection arthroplasty. It is our hope that continued follow-up and future reports will provide insight into a successful strategy for treatment of fungal infections in TEA.
